# Implications of the anti-aging efficacy of BaZi Bu Shen capsules for human longevity and development

**DOI:** 10.3389/fragi.2025.1611959

**Published:** 2025-10-02

**Authors:** Zuohuan Meng, Wenyuan Ma, Xinke Zhao

**Affiliations:** ^1^ Institute of Traditional Chinese Medicine, The Third Affiliated Hospital of Gansu University of Chinese Medicine, Baiyin, China; ^2^ Gansu University of Chinese Medicine, Lanzhou, China

**Keywords:** Bazi Bu Shen capsules, anti-ageing drug, future of mankind, traditional Chinese medicine, DNA methylation age

## Abstract

At present, there are no approved drugs for the treatment of aging, but a number of studies have reported related anti-aging drugs. As an innovative traditional Chinese medicine (TCM) that has been on the market for many years to treat waist and knees soreness, dizziness, tinnitus, fatigue, and cold limbs caused by insufficient kidney-yang, “Bazi Bushen Capsules (BZBS)” has also made a series of new and remarkable achievements in anti-aging research, which is expected to become the first new drug approved by the National Medical Products Administration (NMPA) for anti-aging indications, thus opening up new ideas for the medical field to treat aging. In the context of anti-aging, the general social phenomenon expected to be effectively caused is that people will enter a new era of longevity, and most countries will move from the current aging society to the aged society and even into the super-aging society in the near future. This remark provides forward-looking and guiding suggestions for future research on the relationship between longevity, economy and social protection.

## Highlights

• BZBS has good anti-aging efficacy.
• BZBS could be the first TCM approved by authorities for the treatment of aging.
• Reflections on social development and human civilization in the era of longevity.

## 1 Introduction

Longevity and wellbeing have always been the timeless universal desires and pursuits of people. The significance of longevity is not limited to the length of biological survival, but it is deeply intertwined with various aspects of human physical and mental health, social roles, and personal value realization. The understanding of longevity today remains largely consistent with that of 3500 years ago, without significant breakthroughs. The *Commentary of Zuo* from 3500 ears ago stated that the “upper limit of longevity is 120 years, middle longevity is 100 years, and lower longevity is 80 years” The modern scientifically accepted explanation for human lifespan is based on the cellular aging theory proposed by American scholar Leonard Hayflick and others, which calculates the maximum human lifespan as: cell division times (50 times) × cell division cycle (approximately 2.5 years) = maximum human lifespan (120 years) (Hayflick, 2003).

The greatest enemy of longevity is “accelerated aging,” which refers to the premature appearance of various aging symptoms compared to normal individuals. accelerated aging prevents human life expectancy from reaching the natural maximum, leading to prolonged periods of unhealthy and sub-healthy fatigue. Therefore, accelerated aging is currently a pressing global issue that needs to be addressed.

Traditionally, the treatment of accelerated aging has been extremely complex and difficult, with causes and mechanisms constrained by factors such as congenital parental genetic factors, dietary habits, bodily disorders, environment, cultural cultivation, work-life balance, emotional regulation, and unexpected anomalies. In TCM, aging is viewed as a decline in Jing (essence), Qi (energy), and Shen (spirit) (Wang and Unschuld, 2016).

Today, there is a heightened focus on aging. In terms of regulations, the 11th edition of the “International Classification of Diseases”, released in 2022, added the classification of aging, indicating that aging is a treatable disease, elevating it to an international level for response (World Health Organization, 2022a). In terms of essence, the World Health Organization made further complementary adjustments to the biological aspects of aging in 2022, pointing out that aging is the cumulative effect of various cellular and molecular damages over time, leading to gradual decline in mental and physical abilities, increased risk of diseases, and ultimately death (World Health Organization, 2022b).

Aging is a process in which molecular damage to cells accumulates at the biological level with increasing age. It is currently regarded as an “intervenable disease state” that causes a decline in physical and mental adaptability and an increase in the risk of disease and death (World Health Organization, 2021). In recent years, breakthroughs have been made in the field of cellular aging research: Bi et al. (2025) found that miR-302b in exosomes derived from human embryonic stem cells (hESC-Exo) can reverse the proliferative arrest of senescent cells in mice, achieving functional recovery of senescent cells in the model (Zhao H. et al., 2024); constructed p16-tdT fluorescent reporter gene mice through a dual homologous recombinase system and designed Sn-pTracer and Sn-cTracer genetic systems, confirming that p16^Ink4a^ + endothelial cells and macrophages play different roles in liver injury repair and that the senescent state is reversible (Zhang et al., 2024); created a PanSci of mammalian aging covering more than 20 million cells, revealing that early aging is mainly characterized by a decrease in the number of key cells in tissues such as fat and muscle, while late aging is characterized by systemic inflammation caused by the expansion of immune cells (Wu et al., 2024); elucidated the mechanism by which the STING-IRF3-RB signaling axis initiates cellular senescence, found that the innate immune cGAS-STING pathway activates IRF3 and forms a nuclear complex with RB to block the cell cycle and induce senescence, and confirmed that CDK4/6 inhibitors can alleviate the exacerbation of liver fibrosis caused by IRF3 deficiency. These studies provide a theoretical basis for the development of anti-aging therapies. At present, although there are no approved drugs for aging, related research continues to advance.

## 2 Two star drugs with anti-ageing properties

In recent years, an important breakthrough in anti-aging research has been the β-nicotinamide mononucleotide (NMN), which has already entered the market as a dietary supplement in the United States and other regions and has shown promising basic research data in anti-aging. Recently, another long-standing product on the market, “BZBS”, which treats lumbar and knee soreness due to kidney yang deficiency, fatigue and forgetfulness, dizziness, tinnitus, cold limbs, and physical weakness, has achieved a series of remarkable new advancements in the field of anti-aging research. It deserves our attention and scrutiny.

## 3 Recent anti-aging research on BZBS

BZBS is brown powder with a light smell and slightly bitter taste (Bazi Bushen Capsule, 2025). It is an innovative TCM developed based on establishing the therapy of “tonifying kidney and replenishing essence, regulating yin and yang, warming and supporting vitality, and nourishing body and spirit” according to the common pathogenesis of premature aging and aging-related diseases, “deficiency of kidney essence, deficiency of vitality, and depletion of body and spirit” (Ji et al., 2024).

The anti-aging efficacy of BZBS originates from the synergistic effects of multiple herbal components and their bioactive compounds, which delay organismal aging through multiple aspects such as anti-oxidation, endocrine regulation, inflammation inhibition, immune enhancement, organ function protection, and metabolic and intestinal health regulation (Li H. R. et al., 2022). Ingredients like flavonoids from Cuscuta chinensis, polysaccharides from Lycium barbarum, lignans from Schisandra chinensis, and anthocyanins from Rubus idaeus exhibit potent antioxidant effects. They can scavenge free radicals, activate the Nrf2/ARE pathway, and induce the expression of antioxidant enzymes, thereby reducing oxidative damage, improving mitochondrial function, and delaying cellular senescence (Dong and Wei, 2024; Xu et al., 2021; He et al., 2015; Zhao et al., 2021; Pan et al., 2024). Osthole from Cnidium monnieri, sulfur compounds from Allium tuberosum, and flavonoids from Cuscuta chinensis maintain the function of the hypothalamic-pituitary-gonadal axis by regulating estrogen and androgen levels, preventing osteoporosis and improving reproductive function (Wu et al., 2022; Zhang et al., 2015; Zhang and Wei, 2020; Wei et al., 2020). Tannins from Rosa laevigata inhibit the NF-κB pathway to reduce the release of inflammatory factors (Xu et al., 2010), while polysaccharides from Lycium barbarum enhance the activity of T cells and macrophages, boosting immunity and delaying the senescence of immune cells (Liu et al., 2025). Additionally, lignans from Schisandra chinensis protect the liver and kidneys (Ai et al., 2018), icariin from Epimedium promotes bone formation (Xin et al., 2020), verbascoside from Cistanche deserticola regulates the neuroendocrine axis to enhance stress resistance and promote neural stem cell proliferation (Lu et al., 2023; Li J. et al., 2022). Raspberry ketone improves glucose metabolism (Zhao L. J. et al., 2024), and polysaccharides from Rosa laevigata act as prebiotics to regulate intestinal flora and maintain intestinal barrier function (Zhang et al., 2023). These components work together through multiple targets and pathways to achieve systematic intervention in the aging process and exert comprehensive anti-aging effects.

Pharmacological studies have revealed that BZBS can improve the appearance of aging in mice, inhibit the formation of atherosclerosis, and improve cardiac function; BZBS improves learning, memory and cognitive ability by improving neural function; BZBS can improve osteoporosis and muscle function; BZBS can increase the number and quality of sperm and increase sex hormone levels; its mechanism is related to up-regulating the levels of sirtuin protein 6 (SIRT6) and telomerase reverse transcriptase, down-regulating the levels of aging-related proteins p53, p16 and p21, and reducing inflammation and oxidative reactions (Li H. R. et al., 2022; Ji, 2022). Furthermore, BZBS can reduce the levels of pro-inflammatory cytokines IL-1β, IL-2, IL-12, p70, IFN-γ and TNF-α in the serum of naturally aging mice, and increase the levels of anti-inflammatory factors IL-4 and IL-10, indicating that BZBS plays the role of regulating immune homeostasis and inflammation level of the body and reducing the aging of immune cells (Wang T. et al., 2023; Song et al., 2023).

Preclinical studies of BZBS also show that it exhibits broad biological activities in multiple systems. *In vitro* experiments indicate that the compound formula can enhance the antioxidant capacity of endothelial cells and reduce oxidative damage by activating the Nrf2/ARE pathway (Mao et al., 2023). In D-galactose-induced senescent mouse models, it can regulate telomere length and the p53/p21 pathway, delay cellular senescence, and improve cognitive and motor functions (Ji et al., 2022). In Alzheimer’s disease model mice, BZBS can reduce Aβ deposition and tau protein phosphorylation, and activate Sirt1/PGC-1α to improve mitochondrial function (Lin et al., 2022). In hyperlipidemia animal models, it can regulate blood lipids, inhibit atherosclerosis and vascular inflammation, and enhance myocardial energy metabolism (Huang et al., 2020). Additionally, it has reproductive-promoting effects, upregulating the expression of testosterone synthesis-related proteins to improve sperm quality, while enhancing immune function and inhibiting inflammatory factor release (Ma et al., 2025). It also demonstrates effects in improving insulin resistance and protecting the kidneys and liver in diabetic and liver injury models (Li et al., 2021). In summary, BZBS has multi-target and multi-effect regulatory effects, providing an important basis for subsequent clinical research.

**FIGURE 1:**
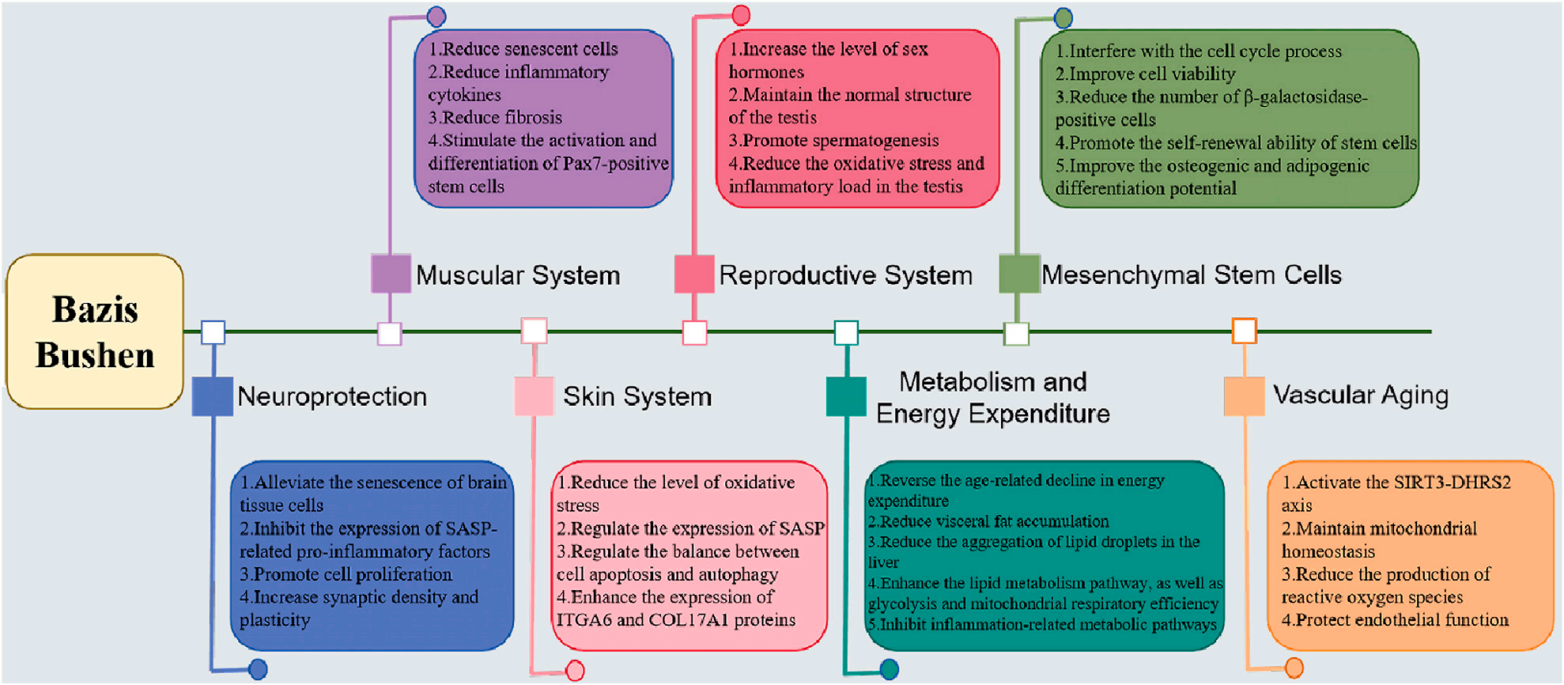
The multi-target anti-aging intervention diagram of BZBS.

BZBS capsules intervene in the aging of multiple tissue systems such as muscles, skin, energy metabolism, reproductive system, and vascular endothelium through the synergistic effects of multiple targets and pathways, as shown in Figure 1. In terms of muscle aging, it can promote muscle cell protein synthesis and mesenchymal stem cell (MSC) proliferation by activating the PI3K/Akt/mTOR and Wnt/β-catenin pathways, while inhibiting the NF-κB inflammatory pathway and mitochondrial dysfunction to reduce muscle degradation. For skin aging, the drug can activate the Nrf2/HO-1 antioxidant system, scavenge ROS damage, regulate the TGF-β/Smad signal to promote collagen production, and inhibit MMPs activity to maintain skin structural stability (Xu et al., 2024; Cheng et al., 2025). In terms of energy metabolism, it improves insulin sensitivity and enhances mitochondrial biogenesis and metabolic homeostasis through the AMPK/mTOR and SIRT1/PGC-1α pathways (Mao et al., 2023; Mima et al., 2023). For the reproductive system, Bazibu Shen capsules can inhibit germ cell apoptosis, regulate the function of the hypothalamic-pituitary-gonadal axis, and maintain sex hormone levels (Mao et al., 2023; Jun et al., 2024). In addition, it can upregulate telomerase activity, inhibit DNA damage, and maintain the genomic stability of MSCs (Zhang Y et al., 2024; Wang Y. et al., 2023). In vascular endothelium, the drug promotes vasodilation through the eNOS/NO pathway, inhibits AGEs/RAGE-mediated oxidative stress and inflammatory response, and improves microcirculatory function (Lu et al., 2023; Mao et al., 2023; Jun et al., 2024). In summary, BZBS capsules achieve systematic intervention in anti-aging by regulating key molecular nodes such as SIRT1, Nrf2, and AMPK, providing a scientific basis for the clinical application of traditional Chinese medicine in anti-aging.

Jun et al. (2024) figured out that BZBS improved premature aging symptoms, quality, and frailty scores of life in a multicenter, parallel, randomized, double-blind, placebo-controlled clinical trial (Clinical Trial Number: ChiCTR2200058262) of 530 participants. As well as improved FTSST (Five-times-sit-to-Standtest), motor function: balance tests, grip strength, muscle mass, and walking speed, and enhanced telomerase activity in elderly subgroups of patients. Telomerase activity is the medical community’s gold standard for maintaining telomere stability and resisting aging. Furthermore, the study found that BZBS is safe and well-tolerated.

This research differs from most previously published preclinical studies or basic clinical investigations. The significance of this study lies in its potential to provide high-level evidence-based medical support for BZBS’s application to add the indication “improvement of age-related functional decline”. Should the study design be rigorous, endpoints clearly defined, and results positive, it could propel BZBS toward its goal of becoming an “anti-aging medication”. Prior to its market entry, drug-related pharmacological and toxicological studies were completed in accordance with current regulations such as the Provisions of Drug Registration (2020), Drug Administration Law of the People’s Republic of China (2019), and Law of the People’s Republic of China on Traditional Chinese Medicine (2017). Based on this research, it is feasible to apply for a new anti-aging indication. The BZBS is expected to be the first CFDA-approved new medication for treating aging, introducing a novel approach to medical treatment for aging. These findings underscore the potential importance of BZBS.

In addition, let’s review an important preclinical study on the anti-aging effects of BZBS. Mao et al. (2023) conducted a 3-month BZBS treatment experiment on male C57BL/6 J mice, and three significant results are worth noting. First, the cognitive abilities of the mice were tested using the Barnes maze testing method, and their motor skills were tested using the Rotarod testing method. The BZBS group showed improvement compared to the control group, and the difference was significant. Secondly, using the “gold standard” indicator of aging, DNA methylation, as a marker, it was found that the BZBS treatment group successfully reversed age-dependent methylation decline, especially in promoter regions, with a very significant effect. By further identifying 602,087 differential methylated regions (DMRs), the study preliminary confirmed that DMRs are critical functional areas of the genome, and there were significant differences in the “young vs. aged” paired groups. Lastly, a noteworthy finding was that the DNA methylation age of the elderly mice receiving BZBS treatment was significantly younger, by approximately 21 weeks, compared to the control group. To this end, the research team has reason to believe that BZBS plays a significant role in extending a healthy lifespan.

Healthcare Exclusive (a platform for recording and observing major business events in the industry, presenting the complexities and conflicts of the industry, and providing insight into cutting-edge industrial trends) published a report on April 27th, entitled “Anti-aging Track with 100 Billion, the Birth Place of the next “Blockbuster Drug”, reporting on the BZBS’s role of prolonging healthy life-span. It was revealed in this report that according to the latest anti-aging research results of TCM with an investment of more than RMB100 million released by the 3rd Anti-Aging Conference of TCM sponsored by World Federation of Chinese Medicine Societies (WFCMS), BZBS group could prolong the longest life span of mice by more than 37 months and the experiment is still in progress; all the mice in the natural aging group died at the age of 30 months and those in the NMN group died at the age of 32 months. The BZBS group showed significantly better outcomes compared to both the natural aging group and the NMN group. Detailed data from this study are still being compiled, and we look forward to the formal publication of the original academic paper, which will provide more rigorous evidence (Caifuhao eastmoney, 2023).

The importance of BZBS in anti-aging lies not only in its efficacy and safety but also in its ease of oral administration, which enhances patient compliance and quality of life.

## 4 Limitations and prospects

In spite of this, the research on anti-aging of BZBS still needs to be further improved. First of all, in view of the synergistic action of TCM with multi-components and multi-targets, it is necessary to further study the mechanism and specific action pathway of its anti-aging, which may contribute to a broader understanding and optimization of the effect of this new therapy.

Secondly, anti-aging research is a subject in initial stage compared with other common diseases, lack of existing reference basis and need long-term exploration in many aspects, such as the BZBS anti-aging theoretical innovation “qi collaterals doctrine and essence-qi-spirit theory” and technological innovation “to construct multi-modal model and multi-level evaluation index system to creatively introduce stress accelerated aging model, *etc.*,” (Mao et al., 2023; Jun et al., 2024).

Finally, it is expected that the common social phenomenon caused by effective anti-aging is that people have entered a new era of longevity, resulting in most countries from the current aging society to the aged society, and even into the hyper-aging society in the near future. This situation may pose a series of serious challenges to the economic and social security system. Without effective social change and material support, the superposition of longevity and artificial intelligence will be a merciless curse and strangulation on the poor, as described in The 100-Year Life: Living and Working in an Age of Longevity.

## 5 Conclusion

In conclusion, the success of BZBS in early studies suggests that multi-target therapies from traditional medicine could play a role in extending healthy lifespan. As we enter an era where living past 100 may become common, it is imperative to continue rigorous research on such interventions and to prepare our healthcare systems for the coming longevity revolution.

## Data Availability

The original contributions presented in the study are included in the article/supplementary material, further inquiries can be directed to the corresponding authors.

## Abbreviations

TCM, traditional Chinese medicine; BZBS, Bazi Bushen Capsules; NMPA, National Medical Products Administration; NMN, β-nicotinamide mononucleotide; DMRs, differential methylated regions; Sn-pTracer, pulse-chase tracing; Sn-cTracer, Cre-based tracing and ablation; PanSci, Pan-genomic Sciences; cGAS-STING, cGAMP synthase-Stimulator of Interferon Genes; IRF3, Interferon Regulatory Factor 3; RB, Retinoblastoma protein; CDK4/6, Cyclin-dependent kinases four and 6; Nrf2/ARE, NF-E2-related factor 2/Antioxidant Response Element; Aβ, Amyloid-beta; tau, Microtubule-associated protein tau; Sirt1/PGC-1α, Sirtuin 1/Peroxisome proliferator-activated receptor gamma coactivator 1-alpha; PI3K/Akt/mTOR, Phosphoinositide 3-Kinase/Protein Kinase B/mammalian Target of Rapamycin; Wnt/β-cateninMSC, wingless Int/beta-catenin Mesenchymal Stem Cells; NF-κB, kappa-light-chain-enhancer of activated B cells; HO-1, Heme oxygenase-1; ROS, Reactive Oxygen Species; TGF-β/Smad, Transforming growth factor beta/Sma and Mad related proteins; MMPs, Matrix metalloproteinases; AMPK/mTOR, AMP-activated protein kinase/mechanistic target of rapamycin; SIRT1/PGC-1α, sirtuin one/peroxisome proliferator-activated receptor gamma coactivator 1-alpha; eNOS/NO, endothelial Nitric Oxide Synthase/Nitric Oxide; AGEs/RAGE, Advanced Glycation End-products/Receptor for Advanced Glycation End-products.
